# Overcoming treatment-resistant depression with machine-learning based tools: a study protocol combining EEG and clinical data to personalize glutamatergic and brain stimulation interventions (SelecTool Project)

**DOI:** 10.3389/fpsyt.2024.1436006

**Published:** 2024-07-17

**Authors:** Mauro Pettorruso, Giorgio Di Lorenzo, Beatrice Benatti, Giacomo d’Andrea, Clara Cavallotto, Rosalba Carullo, Gianluca Mancusi, Ornella Di Marco, Giovanna Mammarella, Antonio D’Attilio, Elisabetta Barlocci, Ilenia Rosa, Alessio Cocco, Lorenzo Pio Padula, Giovanna Bubbico, Mauro Gianni Perrucci, Roberto Guidotti, Antea D’Andrea, Laura Marzetti, Francesca Zoratto, Bernardo Maria Dell’Osso, Giovanni Martinotti

**Affiliations:** ^1^ Department of Neurosciences, Imaging and Clinical Sciences, Università degli Studi G. D’Annunzio, Chieti, Italy; ^2^ Department of Mental Health, ASL02 Lanciano-Vasto-Chieti, Chieti, Italy; ^3^ Institute for Advanced Biomedical Technologies (ITAB), “G. d’Annunzio” University of Chieti-Pescara, Chieti, Italy; ^4^ Laboratory of Psychophysiology and Cognitive Neuroscience, Chair of Psychiatry, Department of Systems Medicine, University of Rome Tor Vergata, Rome, Italy; ^5^ Institute of Hospitalization and Care With Scientific Character (IRCCS) Fondazione Santa Lucia, Rome, Italy; ^6^ Department of Biomedical and Clinical Sciences Luigi Sacco and Aldo Ravelli Center for Neurotechnology and Brain Therapeutic, University of Milan, Milano, Italy; ^7^ Centre for Behavioural Sciences and Mental Health, Istituto Superiore di Sanità, Rome, Italy; ^8^ Psychopharmacology, Drug Misuse and Novel Psychoactive Substances Research Unit, School of Life and Medical Sciences, University of Hertfordshire, Hatfield, United Kingdom

**Keywords:** transcranial magnetic stimulation (rTMS), esketamine nasal spray, machine-learning (ML) algorithms, treatment resistant depression (TRD), endophenotypes

## Abstract

Treatment-Resistant Depression (TRD) poses a substantial health and economic challenge, persisting as a major concern despite decades of extensive research into novel treatment modalities. The considerable heterogeneity in TRD’s clinical manifestations and neurobiological bases has complicated efforts toward effective interventions. Recognizing the need for precise biomarkers to guide treatment choices in TRD, herein we introduce the SelecTool Project. This initiative focuses on developing (WorkPlane 1/WP1) and conducting preliminary validation (WorkPlane 2/WP2) of a computational tool (SelecTool) that integrates clinical data, neurophysiological (EEG) and peripheral (blood sample) biomarkers through a machine-learning framework designed to optimize TRD treatment protocols. The SelecTool project aims to enhance clinical decision-making by enabling the selection of personalized interventions. It leverages multi-modal data analysis to navigate treatment choices towards two validated therapeutic options for TRD: esketamine nasal spray (ESK-NS) and accelerated repetitive Transcranial Magnetic Stimulation (arTMS). In WP1, 100 subjects with TRD will be randomized to receive either ESK-NS or arTMS, with comprehensive evaluations encompassing neurophysiological (EEG), clinical (psychometric scales), and peripheral (blood samples) assessments both at baseline (T0) and one month post-treatment initiation (T1). WP2 will utilize the data collected in WP1 to train the SelecTool algorithm, followed by its application in a second, out-of-sample cohort of 20 TRD subjects, assigning treatments based on the tool’s recommendations. Ultimately, this research seeks to revolutionize the treatment of TRD by employing advanced machine learning strategies and thorough data analysis, aimed at unraveling the complex neurobiological landscape of depression. This effort is expected to provide pivotal insights that will promote the development of more effective and individually tailored treatment strategies, thus addressing a significant void in current TRD management and potentially reducing its profound societal and economic burdens.

## 1 Background

It is imperative to improve our therapeutic strategies and provide optimal treatment options for depression. Major Depressive Disorder (MDD) is a substantial contributor to global disability, affecting more than 300 million people (1). Multiple lines of evidence suggest that MDD may stem from various pathophysiological changes (2), including disruptions in glutamatergic function (3). A significant challenge arises as approximately 30-50% of MDD patients exhibit inadequate responses to initial treatment approaches (4). Consequently, these individuals endure distressing symptoms for extended periods, with a significant portion developing treatment-resistant depression (TRD). TRD is operationally defined as the lack of a substantial therapeutic response after two antidepressant trials that are deemed adequate in both duration (specifically, a minimum of 4-6 weeks) and dosage (5). Studies have shown that individuals with TRD have reduced glutamate levels in prefrontal regions (6).

Recently, two rapid-acting interventions gained approval to address TRD: glutamatergic pharmacotherapies, such as esketamine nasal spray (ESK-NS), and non-invasive brain stimulation, specifically repetitive transcranial magnetic stimulation (arTMS), with accelerated protocols being able to exert similar antidepressant effectiveness to standard protocols with a reduced timeframe (7, 8). Both treatments require a significant time investment and are administered in specialized settings, but there is currently insufficient data guiding the choice between them. rTMS can locally modify cortical excitability in specific brain regions, inducing changes in brain circuits typically underactive in MDD (9). In contrast, ESK-NS acts on glutamatergic ionotropic N-methyl-D-aspartate (NMDA) receptors, transiently increasing glutamate release (10). The challenge of identifying personalized interventions for TRD and MDD remains a significant concern, with the absence of tools able to guide treatment selection as a prominent issue. Coupling effective treatments with suitable patients reduces costs, chronicity, and avoidable suffering (4).

Addressing the “treatment-selection” problem requires a deeper understanding of biomarkers in depression: objectively measurable characteristics reflecting underlying biological processes that contribute to heterogeneity of the MDD subtype and predict the therapeutic response (11–13). Resting-state electroencephalography (EEG), a neuroimaging technique known for its high temporal resolution, appears to be a promising approach for response prediction in depressive illness (14). It is a valuable tool to explore neural biomarkers associated with TRD, offering information on neural activity alterations and functional connectivity related to depression. Evidence suggests that EEG-derived biomarkers, such as alpha band asymmetry, altered EEG resting-state B microstate, or EEG functional connectivity patterns, could accurately help predict treatment outcomes (15–17).

Peripheral blood-based biomarkers, such as markers of systemic inflammation (including interleukines: IL-6, IL-8, IL-2p70) and hormone levels (thyroid-stimulating hormone, cortisol, norepinephrine) can also aid in subtyping MDD and predicting treatment response (18).

These biomarkers are easy to measure and have significant potential for practical implementation in routine clinical practice. Additionally, computational phenotyping, which generates research-grade profiles based on clinical presentation and computer-executable algorithms, contributes to a comprehensive understanding of personalized treatment approaches (19).

Machine learning (ML), a subset of artificial intelligence, encompasses diverse algorithms capable of building predictive models based on specific datasets (20). These algorithmic approaches aim to reveal fundamental principles underlying observations without explicit instructions, extracting structured knowledge from extensive datasets (21). A recent review demonstrates that ML technologies and data analytics can be applied at various stages of the patient journey, including detection and diagnosis, prognosis, treatment selection and optimization, outcome monitoring and tracking, and relapse prevention. Furthermore, data-driven ML approaches can identifying subtypes of symptoms and cognitive deficits, enabling model-based phenotyping (22). In this regard, significant progress has been made in the field of oncology. Specifically, it has become possible to robustly predict treatment responders and non-responders by using network-based biomarker expression levels in patients with melanoma, metastatic gastric cancer, and bladder cancer treated with immune checkpoint inhibitors targeting the programmed cell death 1/programmed cell death ligand 1 axis (23). On the other hand, integrating ML methods with extensive electronic health record databases has the potential to facilitate personalized psychiatry (22).

In this area, to fill the gap between the largely unmet needs of TRD and the enormous potential that has been opened by available innovations (e.g., neuroscience techniques, artificial intelligence methods, and advanced therapeutics), computerized tools can be developed, integrating clinical, neurophysiological, and peripheral data to guide treatment selection. These machine-learning methods could overcome the difficulty of treating TRD and its devastating consequences.

This study aims to develop and preliminarily validate a computational system that integrates clinical, electroencephalographical, and peripheral marker data, thus creating a tool to inform the treatment of Treatment-Resistant Depression (TRD) called “SelecTool”. This tool is designed to support clinical decision-making by helping select personalized, tailored interventions. Using a machine-learning analysis of multi-channel data, the SelecTool will guide treatment selection towards ESK-NS or arTMS. This manuscript delineates the study protocol of the SelecTool project, a translational, multicentric investigation encompassing two distinct phases that aim to develop a machine-learning based tool to help guide clinicians in managing TRD.

## 2 Methods/design

### 2.1 Study design and settings

**Figure 1:**
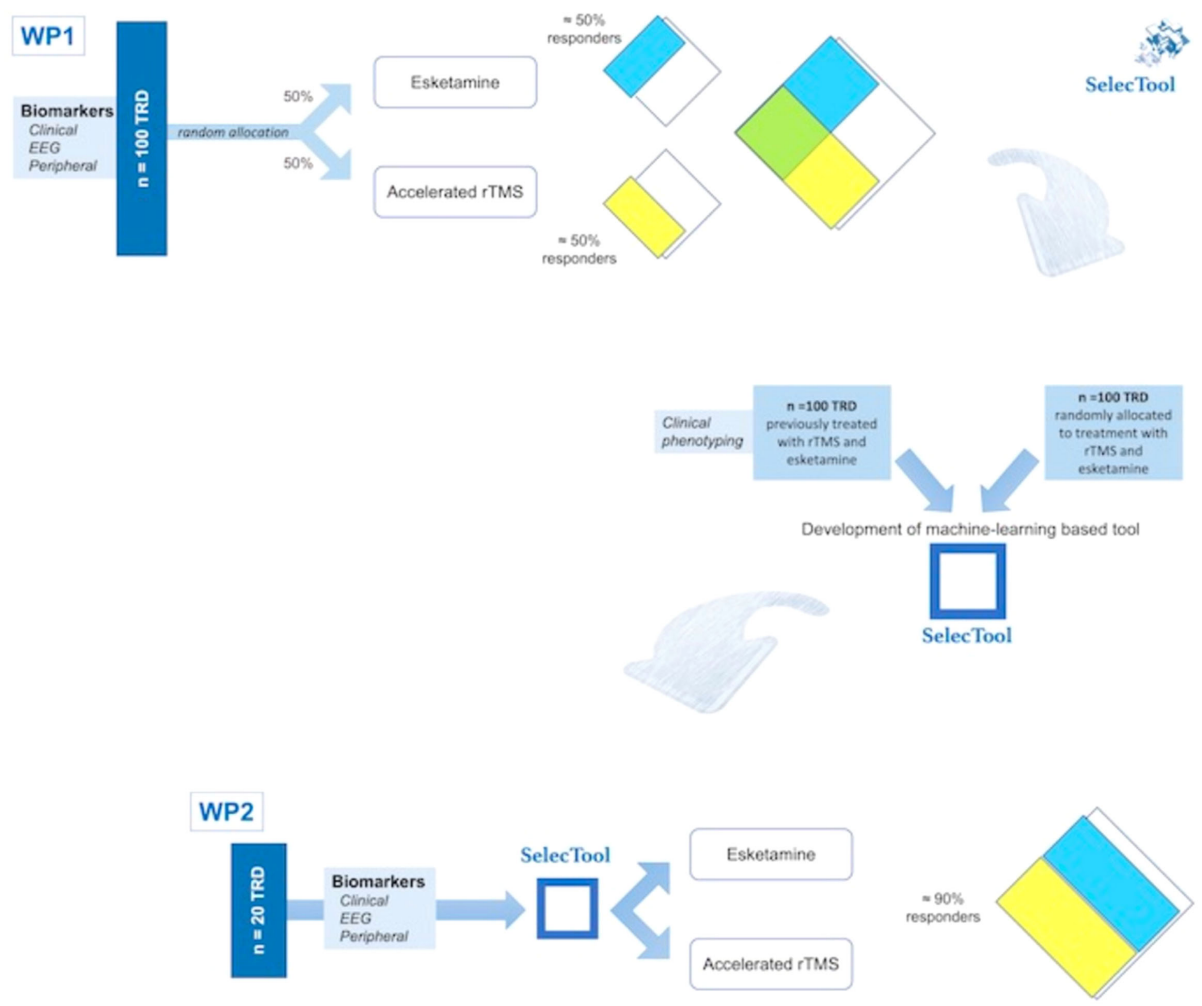
Flow chart of the study protocol describing the two different phases.

The project comprises two phases (**Figure 1**). The first (WP1; see **Figure 1**) involves the development of SelecTool for treatment orientation towards ESK-NS and arTMS by creating a machine-learning system. This phase includes:

• Prospective evaluation of clinical, electrophysiological, and peripheral biomarkers to predict the antidepressant response to ESK-NS and arTMS (n = 100).

• Integration of the above data with those previously collected from subjects with TRD treated with ESK-NS (n = 50) and arTMS (n = 50).

• Training a computerized system to develop a machine-learning-based tool that guides the treatment selection.

The subsequent stage (WP2; see **Figure 1**) focuses on the pilot validation of the ESK-NS and arTMS prescription using SelecTool, including the proof-of-concept estimation of SelecTool’s accuracy in an independent cohort (n = 20; out-of-sample validation). In this step, the identified biomarkers guiding treatment will be integrated into the SelecTool model as input data. Using the SelecTool’s output, individuals will undergo nonrandomized assignment to ESK-NS or arTMS interventions. Therefore, the accuracy in determining an increase in the number of responders to treatment will be estimated and compared with the response rates observed in random assignment.

### 2.2 Sample size and eligibility criteria

One-hundred and twenty subjects (WP1: 100 subjects; WP2: 20 subjects) who are diagnosed with major depressive episode (both during the course of MDD or bipolar disorder) according to the Diagnostic and Statistical Manual of Mental Disorders, 5th edition (24), will be recruited by three Research Units: the ‘G. d’Annunzio’ University of Chieti, the University of Milano, and the Tor Vergata University of Rome.

The inclusion criteria will be the following: age between 18 and 65; current major depressive episode within at least the past month; TRD, defined as the absence of clinical response despite two or more treatments with antidepressants at adequate doses for 4-6 weeks (5); current stable psychopharmacological therapy for at least 1 month.

The exclusion criteria will be the following: presence of severe organic or neurological comorbidities, any substance use disorder (except nicotine dependence) in the past 6 months, intellectual disability or decline (Mini-Mental State Examination, MMSE < 26); uncontrolled systemic hypertension (specific for safety of ESK-NS treatment); presence of a positive history of seizures in the patient’s history or a first-degree relative (specific for arTMS safety); pregnant and postpartum women.

To refine the processing precision of the tool, we will consolidate the dataset from participants enrolled in the study with clinical data gathered retrospectively. These supplemental data will be obtained from a dedicated TRD dataset, which includes information from subjects who met identical inclusion and exclusion criteria and underwent prior treatment in our centers with ESK-NS (n = 50) and arTMS (n = 50).

### 2.3 Study schedule

The enrolled patients will undergo a comprehensive clinical examination as the initial step. Electrophysiological (EEG recordings) and peripheral biomarkers will be collected during this process. The administration of ESK-NS or arTMS will be determined according to the groupings of participants. Specifically, in the first phase, subjects will be randomly assigned to arTMS or ESK-NS. In the second phase, the extracted treatment-orienting biomarkers will be introduced in the SelecTool model as input data. Based on the SelecTool output, subjects will be assigned to the ESK-NS or arTMS interventions.

Baseline and 1-month follow-up assessments will include neuropsychological and psychiatric evaluations, behavioral assessments, neurophysiological data acquisition, and the collection of peripheral biomarkers.

After one month, the clinical response will be measured by a blind rater based on the MADRS score (> 50% reduction).

### 2.4 Neuropsychological and psychiatric assessment

Subjects will undergo assessments at the screening visit (T0) and one month after initiation of treatment (T1) using a battery of validated psychometric tests (**Table 1**). At baseline, collection of anamnestic data will include sociodemographic factors, the history of depressive illness, treatment history for the current major depressive episode (MDE), comorbidities, lifetime antidepressant trials, augmentation strategies (such as the combined use of mood stabilizers, benzodiazepines, or antipsychotics), and other therapeutic interventions for treating treatment-resistant depression (TRD). These evaluations will be conducted by qualified psychiatrists, residents in psychiatry or clinical psychologists blinded to the treatment assignment. The primary outcome will be assessed in terms of clinical response, measured by the Montgomery-Åsberg Depression Rating Scale (MADRS; score reduction > 50%) (25). Patients will be evaluated for mood (Hamilton Depression Scale; Young Mania Rating Scale) (26), anhedonia (Snaith-Hamilton Pleasure Scale) (27), temperamental aspects (Temperament Evaluation of Memphis, Pisa and San Diego Autoquestionnaire version) (28); Barratt Impulsiveness Scale version 11; Big Five Questionnaire) (29, 30), anxiety (Hamilton Anxiety Scale) (31), alexithymia (Toronto Alexithymia Scale) (32), general psychiatric symptoms (Brief Psychiatric Rating Scale) (33), traumatic experiences (Childhood Trauma Questionnaire) (34), suicide risk (Columbia-Suicide Severity Rating Scale, Beck Hopelessness Scale) (35, 36), and resilience (Connor-Davidson Resilience Scale) (37). Health-related assessments will use the Clinical Global Impression-Severity scale (CGI-S) (38).

**Table 1:** Psychometric assessment at T0 and T1.

Psychometric assessment	
Mood	*Hamilton Depression Scale* *Young Mania Rating Scale*
Anhedonia	*Snaith-Hamilton Pleasure Scale*
Temperamental aspects	*Temperament Evaluation of Memphis, Pisa and San Diego Autoquestionnaire version* *Big Five Questionnaire*
Anxiety	*Hamilton Anxiety Scale*
Alexithymia	*Toronto Alexithymia Scale*
General psychiatric symptomatology	*Brief Psychiatric Rating Scale*
Traumatic experiences	*Childhood Trauma Questionnaire*
Suicide risk	*Columbia-Suicide Severity Rating Scale*
Resilience	*Connor-Davidson Resilience Scale*
Health-related assessments	*Clinical Global Impression-Severity scale Health Status Questionnaire*

### 2.5 Behavioral evaluation

A comprehensive neuropsychological evaluation targeting various cognitive functions will be conducted for all patients. The assessment battery will primarily encompass measures of global cognition (MMSE) (39), attention (sustained spatial attention, Trail Making Test-A [TMT-A]; divided spatial attention, TMT-B; cognitive flexibility, TMT-AB) (40), short- and long-term episodic memory (Babcock Memory test) (41), and executive function (Frontal Assessment Battery) (42).

### 2.6 Neurophysiological data

At T0 and T1, EEG electrical activity will be acquired utilizing a 64-channel EEG system (eego™mylab; ANT Neuro, Hengelo, Netherlands). Resting-state EEG will be recorded with eyes open and closed. Electrooculography and electrocardiography will also be acquired using additional electrodes. The data will undergo pre-processing to eliminate sections of poor quality and channels with unreliable data. Independent component analysis will be applied to eliminate periodic, non-brain signals. EEG analysis aims to identify pertinent and effective electro-neurophysiological biomarkers (at the channel/scalp, source, and source connectivity levels) indicative of treatment response in TRD (**Table 2**).

**Table 2:** EEG biomarkers to predict treatment response.

**Alpha asymmetry**	Based on the approach-withdraw model (43), this measures relative alpha band activity between brain hemispheres (mainly in frontal regions; higher alpha may reflect lower brain activity). Alpha asymmetry has been proposed as a suitable prognostic biomarker related to anxious subtype and bipolar features (44).
**Microstate abnormalities**	Using polarity-insensitive k-mean clustering, we will segment resting-state high-density EEG data into microstates (45). The proportion, duration, occurrence, and transition of microstates will be studied as potential biomarkers of state and trait abnormalities and as predictors of treatment outcome.
**Rostral anterior cingulate cortex theta activity**	This is a robust marker that predicts greater improvement in selective serotonin reuptake inhibitor-induced depressive symptom (46).
**Subgenual/prefrontal connectivity**	Based on recent findings that suggest that changes in rTMS-induced within-network connectivity are a mediator of treatment response (47), eLORETA linear-lagged connectivity measures of theta (4-7.5 Hz) and alpha (8-13 Hz) frequency will be obtained between the following regions of interest: right and left DLPFC, dorsomedial prefrontal cortex, and subgenual cingulate cortex (as in Iseger et al, 2017).
**Gamma-band power envelope connectivity**	Orthogonalized power envelope correlation will measure EEG source connectivity (48). Large-scale connectivity patterns have been proposed as predictors of placebo/antidepressant outcomes.

### 2.7 Peripheral biomarkers

Blood samples (15 ml) will be collected at T0 and T1 by forearm venipuncture after an overnight fast. These samples will be stored in BD Vacutainer tubes containing ethylenediaminetetraacetic acid. Serum and plasma will be prepared by centrifugation at 1500 rpm for 10 minutes at 4°C. The serum will be stored in 0.5 ml Eppendorf tubes at -80°C until analysis.

Enzyme-linked immunosorbent assays (ELISAs) will be used to assess systemic inflammation and oxidative stress markers, including C-reactive protein, interleukin-1b (IL-1b), IL-5, IL-6, IL-8, and tumor necrosis factor-alpha (TNF-alpha). The levels of cortisol and adrenocorticotropic hormone will be determined using ELISA. Using specific monoclonal antibodies, the levels of TSH, FT3, and FT4 will be determined. Plasma brain-derived neurotrophic factor (BDNF) and proBDNF levels as biomarkers of synaptic integrity and plasticity will be investigated using ultra-sensitive high-performance single-molecule arrays or conventional ELISA.

### 2.8 Treatment administration

During the first phase (WP1), subjects will be randomly assigned in a 1:1 ratio to receive arTMS or ESK-NS. A stratified randomization approach with a four-block size will be implemented to minimize inadvertent bias. Stratification factors will include sex (male, female), age (expected cutoff 50 years old), depression severity (mild/moderate depression, MADRS ≤ 34; severe depression, > 34), and treatment site (Chieti, Milan, and Rome). The randomization process will be carried out by an investigator external to the study.

In the pilot validation phase (WP2), the extracted treatment-guiding biomarkers will be incorporated into the SelecTool model as input data. Subsequently, subjects will be allocated to esketamine or arTMS interventions based on the output of the SelecTool.

All subjects will undergo a comprehensive preliminary visit to assess potential contraindications to treatments. Qualified medical personnel, specifically trained to handle potential side effects and emergencies related to treatments, will administer ESK-NS and arTMS treatments.

Subjects in the ESK-NS group will be administered the drug according to the EMA guidelines (49). It will be supplied in a double-use nasal spray device containing 200 μl of vehicle solution (two sprays), each delivering 28 mg (14 mg ESK-NS base per 100 μl of spray). This dose will be administered twice a week for the first week, followed by 84 mg (three devices) administered twice a week for three weeks, resulting in a total of 1 month of treatment. Before initial administration, patients will be instructed to blow their nose (only before the first device is administered) and then assisted to recline their head 45° (semi-reclined position) during administration to enhance retention of the medication within the nasal cavity. Each ESK-NS session will be conducted by qualified personnel who closely monitor vital parameters (blood pressure, heart rate) before and at 45 and 90 minutes after treatment, following international safety guidelines (50).

Patients in the arTMS group will undergo a 5-day arTMS protocol involving four daily sessions (8). This protocol, developed following the safety guidelines (51) and the principles of accelerated protocols (52), aims to deliver the same number of magnetic pulses as the FDA-approved protocol (53). Stimulation will be performed using a MagPro R30 (MagVenture) system with a B-70 coil targeting the left dorsolateral prefrontal cortex (L-DLPFC), a region approved for TRD treatment (53). The L-DLPFC will be identified using the BEAM F3 method (54), facilitating rapid localization through anthropometric measures. The resting motor threshold will be determined using the evoked potential motor method (55). Each session will adhere to the following parameters, aligning with the FDA-approved standard (53): 10 Hz frequency, 120% resting motor threshold, 40 pulses/train (4 s duration), 26 s inter-train interval, 3000 pulses/session, and a total duration of 35 minutes. This session will be repeated four times within the same day, with a 55-minute interval between sessions (total duration of the cycle session pause: 90 minutes), thus adhering to the accelerated stimulation protocol. The entire protocol will take approximately 5 hours and 5 minutes. Throughout this time, patients will be continuously monitored for side effects. The onset of potential side effects will be evaluated at each stimulation session using a specific and approved scale for rTMS-related side effects (56).

### 2.9 Statistical analysis

Drawing from the existing literature on the efficacy of arTMS and ESK-NS for Treatment-Resistant Depression (TRD), we anticipate a response rate of approximately 50% for each treatment (52, 57). The sample size was determined using the G*Power 3.1 software, taking into account specific parameters: a substantial effect size of predictors (expected Cohen effect size F = 0.4), power 1-beta = 0.80, one-way, four groups (2x2; treatment: ESK-NS, arTMS; responders and non-responders), and a significance level corrected for multiple comparisons (alpha = 0.001). These calculations resulted in a total sample size of n = 144. Considering a possible imbalance in the allocation of responders (10%) and to mitigate possible dropouts (10%), we increased the total sample size to n = 200.

We will develop a machine-learning model to predict the primary treatment outcome and use it for treatment guidance. This model will leverage both neurophysiological data and clinical scores. We also aim to interpret the model and extract the features that influence it. Given the heterogeneous nature of the collected data, an appropriate solution is to opt for ensemble methods, particularly random forest techniques, which have shown suitability for such tasks (19, p. 202) and are relevant for *post hoc* analysis of results. To provide comprehensive insights, our exploration will not be restricted to random forest techniques; we will also investigate other approaches such as neural networks or support vector classifiers. Dealing with missing values in clinical and psychometric tests is a critical concern, and we will address this using advanced techniques such as multivariate imputation (58, 59). The model parameters and performance will be assessed using nested and shuffle cross-validation, which is recognized as optimal to minimize bias in model error estimations (60). The results’ significance will be evaluated using permutation tests, which are acknowledged as the gold standard for statistical assessments of machine-learning algorithms (61).

### 2.10 Ethical issues

This study has received approval from a local Institutional Review Board (C.Et.R.A., approval number: 6/2023). It will follow the principles and recommendations of Good Clinical Practice and the Declaration of Helsinki (World Medical Association, 2013), which offer guidance to physicians engaged in biomedical research with human subjects. The patient will sign the informed consent form, which will be witnessed, dated, and retained by the investigator responsible for recruiting patients into the study.

## 3 Discussion

This research proposal is designed to spearhead an innovative methodology for enhancing clinical decision-making processes in the context of TRD. The aim is to develop and preliminarily validate an advanced computational framework that adeptly consolidates clinical assessments, peripheral biomarkers, and EEG data to address the selection of advanced treatment for TRD. This integration aims to predict treatment outcomes precisely (62), thus facilitating the tailored orientation of therapeutic strategies that reduce unnecessary suffering. To construct this pivotal tool for TRD treatment optimization, we plan to utilize a machine learning algorithm capable of processing complex, multi-dimensional data streams (18, 63).

Machine learning has been employed in the medical sector since the late 1990s, notably in oncology – a principal area of application (20). Within this field, a critical challenge involves the identification of markers that can accurately predict drug responses among diverse groups of cancer patients. A recent study introduced a network-based machine-learning framework capable of generating robust predictions across immune checkpoint inhibitor datasets and pinpointing potential biomarkers (23).

In the area of clinical neurosciences, there is significant potential for benefit from these technological advances, especially considering the nuanced presentation of symptoms characteristic of neurological disorders. A study conducted in 2022 focused on the use of machine learning algorithms to classify subtypes of immune microenvironment and identify unique genes in Alzheimer’s disease. This research highlighted five immune microenvironment-related genes that strongly correlate with pathological markers and reliably predict the disease’s trajectory (64).

The field of psychiatry has also seen considerable advancements through pioneering research efforts. A recent multicenter study applied multimodal machine learning methods, integrating clinical, neurocognitive, structural magnetic resonance imaging, and polygenic risk scores to predict the onset of psychosis in individuals at high clinical risk or with recent-onset depression (65). Furthermore, a recent narrative review investigated the application of machine learning in diagnosing and forecasting schizophrenia, concluding that various machine learning-based models can potentially help healthcare professionals in diagnosing the condition and predicting its clinical presentations and complications (66).

Concerning TRD, a very recent machine learning study has highlighted that characteristics such as profound anhedonia, anxious distress, mixed symptoms, and bipolarity in patients treated with ESK-NS represent factors that predict a positive response and remission. In contrast, the use of benzodiazepines and the severity of depression were associated with delayed responses (67). The levels of accuracy achieved with data exclusively symptom-based do not allow for incorporation into clinical practice and justify the attempt of the SelecTool Project to refine selection methods by integrating other biomarkers.

Given the substantial global health impact of TRD, which doubles the risk of hospitalization and increases the risk of suicide sevenfold compared to treatment-responsive depressed patients (68) our primary objective is to identify treatment approaches that optimize patients’ prospects for recovery. On the one hand, arTMS is a proven intervention for TRD, strongly supported by existing literature, demonstrating response rates of 40–50% and remission rates of 25–30%. (52, 69). On the other hand, in patients treated with ESK-NS, the percentage of remitters has been observed to be less than half (70).

As a result, despite the established antidepressant efficacy of ESK-NS and arTMS, achieving clinical response rates of approximately 50-60% even in real-world studies (8, 71–75), there remains a notable gap in our understanding of their response biomarkers. This proposal is also set to significantly expand our understanding of the complex and heterogeneous nature of the pathophysiology and treatment of MDD. Viewing MDD through the lens of brain connectivity disorders highlights its varied neurobiological foundations, likely related to disparate brain network functionalities (76). Such neurobiological diversity leads to distinct MDD subtypes, each with its unique treatment response profile, particularly to neuromodulation and glutamatergic interventions.

By deepening our understanding of the biomarkers associated with various depression subtypes, including clinical, EEG, and peripheral indicators, we aim to pioneer a patient-centered approach to treatment selection. Given the substantial social, occupational, and physical repercussions associated with TRD, not to mention the increased healthcare costs that make TRD a significant economic burden on healthcare systems (68, p. 201; 77, 78), this research has the potential for considerable social and economic benefits.

In conclusion, this research proposal not only aims to change the approach to treating TRD by leveraging cutting-edge machine learning techniques and comprehensive data analysis, but also aims to shed light on the intricate landscape of the neurobiological underpinnings of depression. Through this endeavor, we anticipate contributing valuable insights that could influence and offer potential advantages for clinical practice, facilitating the development of more effective and personalized treatment regimens. This approach addresses a critical gap in the current management of TRD and potentially alleviating its significant societal and economic impacts.

## Data availability statement

The original contributions presented in the study are included in the article/supplementary material. Further inquiries can be directed to the corresponding author/s.

## Ethics statement

The studies involving humans were approved by Comitato Etico Regione Abruzzo (C.Et.R.A.). The studies were conducted in accordance with the local legislation and institutional requirements. The participants provided their written informed consent to participate in this study.

## Author contributions

MP: Writing – original draft, Writing – review & editing. GD: Writing – original draft, Writing – review & editing. BB: Writing – original draft, Writing – review & editing. Gd: Writing – original draft, Writing – review & editing. CC: Writing – original draft, Writing – review & editing. RC: Writing – original draft, Writing – review & editing. GM(7^th^ author): Writing – original draft, Writing – review & editing. OD: Writing – original draft, Writing – review & editing. GM(9^th^ author): Writing – original draft, Writing – review & editing. AD(10^th^ author): Writing – original draft, Writing – review & editing. EB: Writing – original draft, Writing – review & editing. IR: Writing – original draft, Writing – review & editing. AC: Writing – original draft, Writing – review & editing. LP: Writing – original draft, Writing – review & editing. GB: Writing – original draft, Writing – review & editing. MP: Writing – original draft, Writing – review & editing. RG: Writing – original draft, Writing – review & editing. AD(18^th^ author): Writing – original draft, Writing – review & editing. LM: Writing – original draft, Writing – review & editing. FZ: Writing – original draft, Writing – review & editing. BD: Writing – original draft, Writing – review & editing. GM(22^nd^ author): Writing – original draft, Writing – review & editing.
